# Correction: Teaching medical ethics and medical professionalism in Saudi public and private medical schools

**DOI:** 10.1371/journal.pone.0352133

**Published:** 2026-06-22

**Authors:** The PLOS One Editors

## Notice of Republication

This article [1] was republished on June 29, 2025, to address an issue identified post-publication. An updated version of S2 File is provided with this notice. Please download this article again to view the correct version.

The article’s Data Availability statement is updated to: Partial individual-level data underlying the results are available within the paper and its Supporting Information files. The full individual-level data are restricted and are available on request from the Department of Family and Community Medicine, King Saud University, at FCM@ksu.edu.sa.

## Supporting information

S2 File Underlying data. (XLSX)
