# Supplementary material for: Teaching medical ethics and medical professionalism in Saudi public and private medical schools
Source: PLoS One. 2024 Feb 29;19(2):e0298605. doi: 10.1371/journal.pone.0298605 (PMC10903870; doi:10.1371/journal.pone.0298605)
Supplement: S1 File — (PDF) [file pone.0298605.s001.pdf]

Teaching and assessment of Medical ethics and professionalism in the medical curriculum

Dear Colleagues,
We are a research group from the college of medicine, King Saud University, lead by Prof. Mohammed Al-Rukban, our principal investigator conducting a scientific study.The aim of this study is to determine the nature, content, and methods of medical ethics and professionalism education in Saudi medical schools.We have an approved IRB number (E-21-6075).

Kindly take 5 minutes to answer the following survey that describes your college's status (as a representative of your school). The results will be used for scientific purposes and all of your answers will be confidential.

Thanks for participating! For any questions don't hesitate to contact us:
email: Teaching.Ethics.Research@gmail.com
phone number: 0543071955.

Demographics

1. 1. Name of your medical college?

____

2. 2. At which year the college was established?

____

Medical ethics

3. 3. Is there a department of medical ethics in your medical school?

Check all that apply.

- [ ] Yes
- [ ] No

4. 4. How are the contents related to medical ethics taught in your curriculum?

Mark only ~~one oval~~.
one answer

- ◯ We have a specific course for medical ethics and ethical content embedded into different courses
- ◯ We have only a specific course for medical ethics
- ◯ We have only ethical content embedded into different courses
- ◯ No content related to medical ethics in the curriculum
- ◯ Other: ____________________

5. 5. How many ethic courses are there in the curriculum?

Mark only one oval.

- ◯ 1
- ◯ 2
- ◯ 3
- ◯ more than 3

6. 6. At which year are the medical ethic courses taught ? (You can choose more than one answer)

Check all that apply.

- ☐ Preparatory year
- ☐ First year of medical school
- ☐ Second year of medical school
- ☐ Third year of medical school
- ☐ Fourth year of medical school
- ☐ Fifth year of medical school
- ☐ Internship

7. 7. What is the total credit hours for all medical ethics courses in your school ?

Check all that apply.

- [ ] 0
- [ ] 1
- [ ] 2
- [ ] 3
- [ ] 4
- [ ] 5
- [ ] 6
- [ ] 7
- [ ] 8
- [ ] More than 8

8. 8. Is the medical ethics course compulsory or elective?

Mark only one oval.

- ( ) Compulsory
- ( ) Elective
- ( ) One course is compulsory and one course is elective

9. 9. What is the percentage of staff who teach medical ethics holding a qualification in ethics?

Mark only one oval.

Do you need to add" I do not know" option?

- ( ) 0%
- ( ) 1-25%
- ( ) 26-50%
- ( ) 51-75%
- ( ) More than 75%

10. 10. What are the topics taught in the medical ethics curriculum ? (You can choose more than one answer)

Check all that apply.

- [ ] Brain death
- [ ] Organ transplantation
- [ ] Cosmetic surgery
- [ ] Abortion
- [ ] Terminal care
- [ ] Contraception and sterilization
- [ ] Doctor’s rights
- [ ] End-of-life issues
- [ ] Medical errors
- [ ] Doctor–patient relationship
- [ ] Fiqh of patient’s prayer and fasting
- [ ] Saudi medico-legal system
- [ ] Prophetic medicine
- [ ] Medical necessity Assisted reproduction
- [ ] Communication with medical industry
- [ ] Genetics
- [ ] History of ethics
- [ ] Ethical principles
- [ ] Cross-cultural issues and diverse beliefs
- [ ] Informed consent
- [ ] Confidentiality and privacy
- [ ] Research ethics
- [ ] HIV/AIDS
- [ ] Discrimination in healthcare
- [ ] Immunizations
- [ ] Individual autonomy
- [ ] Equity and equality
- [ ] Patients rights
- [ ] Ethics of interviewing

Other: [ ] ____________________

11. 11. What are the teaching methods used in the medical ethics course? (You can choose more than one answer)

Check all that apply.

- [ ] Lectures
- [ ] Case studies
- [ ] PBL (Problem based learning)
- [ ] Students' presentations

Other: [ ] ______________________

12. 12. What types of assessment methods are used in the medical ethics courses? (You can choose more than one answer)

Check all that apply.

- [ ] Multiple choice questions (MCQs)
- [ ] Short answer questions (SAQ)
- [ ] Objective structured clinical examination (OSCE)
- [ ] Research
- [ ] Assignments

Other: [ ] ______________________

13. 13. What are the challenges and obstacles of teaching medical ethics? (You can choose more than one answer)

What if the student does not perceive any obstacles?

Check all that apply.

- [ ] Ethics curriculum demands a lot of time
- [ ] Unavailable qualified staff
- [ ] Lack of guidelines/resources
- [ ] Unsupportive institutional culture
- [ ] Lack of student interest

Other: [ ] ______________________

Professionalism

14. 14. How are the contents related to professionalism taught in your curriculum ?

Mark only one oval.

- We have a specific course in professionalism and professional content embedded into different courses
- We have only a specific course for professionalism
- We have only professional content embedded into different courses
- No content related to medical professionalism in the curriculum
- Other: ______________________

15. 15. What is the total credit hours for all the professionalism courses at your school?

Mark only one oval.

- 0
- 1
- 2
- 3
- 4
- 5
- 6
- 7
- 8
- More than 8

16. 16. Is the professionalism course compulsory or elective?

Mark only one oval.

- Compulsory
- Elective
- One course is compulsory and one course is elective

17. 17. At which year medical Professionalism course is taught ? (You can choose more than one answer)

Check all that apply.

- [ ] Preparatory year
- [ ] First year of medical school
- [ ] Second year of medical school
- [ ] Third year of medical school
- [ ] Fourth year of medical school
- [ ] Fifth year of medical school
- [ ] Internship

18. 18. What are the topics taught in the professionalism curriculum? (You can choose more than one answer)

Check all that apply.

- [ ] Concepts and principles of professionalism
- [ ] Doctor’s character
- [ ] Inter-professional relationship
- [ ] Examining patients
- [ ] Unprofessional behavior
- [ ] Volunteering and community commitment
- [ ] Communication skills
- [ ] Team work
- [ ] Time management
- [ ] Stress management
- [ ] Management and leadership
- [ ] Breaking bad news
- [ ] Doctor–patient relationship

Other: [ ] ______________________________

19. 19. What are the teaching methods used in the professionalism course?(you can choose more than one answer)

Check all that apply.

- [ ] Lectures
- [ ] Case studies
- [ ] PBL (Problem based learning)
- [ ] Students' presentations

Other: [ ] ____________________

20. 20. What types of assessment methods are used in the professionalism courses ? (you can choose more than one answer)

Check all that apply.

- [ ] Multiple choice questions (MCQ)
- [ ] Short answer questions (SAQ)
- [ ] Objective structured clinical examination (OSCE)
- [ ] Research
- [ ] Assignment

Other: [ ] ____________________

21. 21. What are the challenges and obstacles of teaching professionalism? (You can choose more than one answer)

What if the student does not perceive any obstacles?

Mark only one oval.

- ( ) Lack of time in the curriculum
- ( ) Unavailable qualified staff
- ( ) Lack of guidelines/resources
- ( ) Unsupportive institutional culture
- ( ) Lack of student interest
- ( ) Other: ____________________

This content is neither created nor endorsed by Google.

Google Forms
