# Supplementary material for: Teaching medical ethics and medical professionalism in Saudi public and private medical schools
Source: PLoS One. 2024 Feb 29;19(2):e0298605. doi: 10.1371/journal.pone.0298605 (PMC10903870; doi:10.1371/journal.pone.0298605)
Supplement: Attachment — Submitted filename: Review.docx [file pone.0298605.s003.docx]

Authors have explored an important component of undergraduate medical training and have provided the evidence regarding the current status, which is lacking required uniformity and is deficient in many aspects despite an apparent willingness and effort to enrich the curriculum and training in relation to medical ethics and professionalism

However, there are areas where the study/manuscript needs improvement

1. At the start of result it was mentioned that 28 medical schools participated in the study. But in the same paragraph while discussing presence of ethics departments the count is 24+5=29

Of the medical schools participating in the study, 24 (82.1%) had no medical ethics department, while 5 (17.9%) had a medical ethics department

2. From the results, it appears that ethics and professionalisms are taught either as a course or embedded in other courses or both but separately from each other which may be the case but surely these are also combined under a single course in few if not many medical colleges. Authors need to clarify about this
3. It will be good to mention the year in which the “Pond’s report” and “Tomorrow's Doctors,” were published
4. Authors have mentioned that, “The development of the medical ethics curriculum has evolved over the last century, both in the Kingdom of Saudi Arabia and in other parts of the world”. From the discussion, it is apparent that these developments belong to maximum last 50 years and not full century. It is suggested to replace the phrase ‘last century’ with ‘last 5 decades’
5. More importantly, there is a definite deficiency in literature review. The authors have mentioned about different teaching strategies and assessment tools being employed in medical schools of Saudi Arabia. For example, they mentioned that,

“There have been no changes in the assessment methods used in medical ethics courses since 2013, when it was reported that the use of MCQs was the most popular method used for assessing medical ethics”

Whereas, the following two articles (published in 2016 and 2021) related to teaching and assessing medical ethics and professionalism in a Sudia Arabian medical college are indicating innovations with successful use of “Portfolio Workbook”, both as a teaching modality and as a major component of assessment. It is recommended that authors should go through these papers and include the innovations in their discussion

a. Innovation in ethics and professionalism course: Early experience with portfolio-workbook. Shamim MS, Zubairi NA, Sayed MH, Gazzaz ZJ. J Pak Med Assoc. 2016 Sep;66(9):1149-1153. PMID: 27654737.
b. Systematic development and refinement of a contextually relevant strategy for undergraduate medical ethics education: a qualitative study. Shamim MS, Torda A, Baig LA, Zubairi N, Balasooriya C. BMC Med Educ. 2021 Jan 6;21(1):9. doi: 10.1186/s12909-020-02425-6. PMID: 33407410; PMCID: PMC7786930.
6. Finally, it is recommended that authors should emphasize more in their conclusion about lack of practical assessment tools as compared to dependency on MCQs
